# Indirect calorimetry in the pediatric ICU

**DOI:** 10.1186/cc14659

**Published:** 2015-09-28

**Authors:** Zina Maria Almeida de Azevedo, Daniella B Moore, Luis Fernando P Amendola, Margarida dos Santos Salú, Daniella ML Caixeta, Elza Rosa Pedroso, Djamayna VC Oliveira, Eloane Gonçalves Ramos

**Affiliations:** 1 Universidade Federal Fluminense, Flamengo, Rio de Janeiro, São Paulo, São Paulo, Brazil; 2 Instituto Fernandes Figueira--Fiocruz, Flamengo, Rio de Janeiro, São Paulo, São Paulo, Brazil

## Introduction

Failure to accurately estimate energy requirements may result in an impaired recovery. Overfeeding has been associated with increased carbon dioxide production, respiratory failure, hyperglycemia and fat deposits in the liver, while underfeeding can lead to malnutrition, muscle weakness and impaired immunity.

## Objective

This study aimed to determine the metabolic profile of infant and preschool children submitted to mechanical ventilation in the ICU.

## Methods

A prospective study was carried out in a pediatric ICU in Rio de Janeiro that included children aged from 1 month to 6 years submitted to mechanical ventilation from June 2013 to May 2015. Indirect calorimetry was used to obtain resting energy expenditure (REE) and oxygen consumption (VO_2_) in the first 48 hours of admission. The predicted basal metabolic rate (PBMR) was calculated using the Schofield equation. The metabolic state of each patient was assigned as hypermetabolic (REE/PBMR >110%), hypometabolic (REE/PBMR <90%) or normal (REE/PBMR 90-110%). The ratio of caloric intake to REE was also calculated and ratios of >1.5 and <0.5 were classified as overfeeding and underfeeding respectively.

## Results

A total of 35 infants and 17 preschool children were included. The male/female ratio was 34/18. In respect of severity of sepsis, 19 patients had septic shock, 24 had sepsis, five had severe sepsis and four had systemic inflammatory response syndrome. We observed a high incidence of hypometabolism (88.5%) and a low incidence of normal metabolism (7.7%) and hypermetabolism (3.8%). A low value of VO_2 _was observed in 46.1% of the patients (VO_2 _≤120 ml/minute/m^2^), a normal value in 40.4% (VO_2 _>120 to ≤160 ml/minute/m^2^) and a high value in only 13.5% of the patients (VO_2 _> 160 ml/minute/m^2^). Among the 52 included patients, 18 were fasting at the moment of the examination. The ratio of caloric intake to REE for the remaining 34 patients showed 38.2% overfeeding, 11.8% underfeeding and 50.0% normal feeding.

## Conclusion

Predictive equations do not accurately predict REE in critically ill infants and preschool children, resulting in inadequate feeding. Although hypermetabolism and enhanced energy expenditure are the main clinical features of critical illness in adults, the majority of our patients were found to be hypometabolic which reinforces the need for a different approach between adult and pediatric critically ill patients.

